# Long-term angiographic outcome of stent-assisted coiling compared to non-assisted coiling of intracranial saccular aneurysms

**DOI:** 10.3325/cmj.2015.56.24

**Published:** 2015-02

**Authors:** David Ozretić, Marko Radoš, Goran Pavliša, Zdravka Poljaković

**Affiliations:** 1 Department of Diagnostic and Interventional Radiology, University Hospital Center Zagreb, University of Zagreb School of Medicine, Zagreb, Croatia; 2 Department of Neurology, University Hospital Center Zagreb, University of Zagreb School of Medicine, Zagreb, Croatia

## Abstract

**Aim:**

To compare angiographic result at long-term follow-up, and rates of progressive occlusion, recurrence, and retreatment of stent-assisted coiled (SAC) and non-assisted coiled (NAC) intracranial saccular aneurysms.

**Methods:**

Retrospective evaluation of department records identified 260 patients with 283 saccular intracranial aneurysms who had long-term angiographic follow-up (more than 12 months) and were successfully treated with SAC (89 aneurysms) or NAC (194 aneurysms) at the University Hospital Center Zagreb from June 2005 to July 2012. Initial and control angiographic results in both groups were graded using Roy/Raymond scale, converted to descriptive terms, and the differences between them were evaluated for statistical significance. A multivariate analysis was performed to identify factors related to progression of aneurysm occlusion and recurrence at follow-up, and those related to aneurysm retreatment.

**Results:**

There were more progressively occluded aneurysms in SAC group (38 of 89 aneurysms, 42.7%) than in NAC group (46 of 194, 23.7%) (*P* = 0.002), but there were no significant differences in the rates of recanalization, regrowth, and stable result. Multivariate logistic regression identified the use of stent as the most important factor associated with progressive occlusion (*P* = 0.015, odds ratio 2.22, 95% confidence interval 1.17-4.21), and large aneurysm size and posterior circulation location as most predictive of aneurysm recurrence and retreatment.

**Conclusion:**

The use of stent is associated with delayed occlusion of initially incompletely coiled aneurysms during follow-up, but does not reduce the rate of recurrence and retreatment compared to coiling alone. Long-term angiographic follow-up is needed for both SAC and NAC aneurysms.

The aim of intracranial aneurysm treatment is prevention of rupture. For a long time, neurosurgical procedure of clipping the aneurysm neck for exclusion from circulation was the only option, but the operation could not be performed in a certain number of patients. In the last 20 years an alternative solution for such patients has been endovascular technique of embolization with coils (coiling) (1). International Subarachnoid Aneurysm Trial (ISAT) study showed a better clinical outcome in coiled patients (2), but also worse long-term morphological result and more frequent recurrences (3). Aneurysm recurrence means that risk of rupture is not eliminated, ie, that the goal of treatment is not achieved. This primarily occurs in large and wide-neck aneurysms (4), hence intracranial stents are introduced to “reconstruct” the aneurysm neck and achieve more complete occlusion, thus leading to fewer recurrences (5-7). Moreover, delayed occlusion of initially incompletely filled aneurysms after stent-assisted coiling was reported (8). The aim of this study was to compare initial and control angiographic result and the rates of progressive occlusion, recurrence, and retreatment during long-term follow-up of stent-assisted (SAC) and non-assisted coiled (NAC) aneurysms.

## Methods

### Patient selection

Retrospective evaluation of medical records and image archive at the Department of Diagnostic and Interventional Radiology, University Hospital Center Zagreb identified 489 patients with 529 saccular intracranial aneurysms successfully treated with NAC or SAC from June 2005 to July 2012. There were 260 patients with 283 aneurysms who had long-term angiographic follow-up (more than 12 months) – 194 aneurysms in NAC group and 89 aneurysms in SAC group. Aneurysms treated with balloon-assisted coiling or with flow-diverter stents were excluded from the study. Demographic and clinical data about patients, anatomical data about treated and retreated aneurysms, and control angiographic exams were collected and analyzed after study approval by the Ethics Committees of the University Hospital Center Zagreb and University of Zagreb School of Medicine.

### Treatment method selection

Primary indication for SAC was unfavorable dome-to-neck ratio (width of the sac divided by the length of the neck, if the ratio is lower than 2, coils are more likely to prolapse out of the aneurysm), regardless of the aneurysm neck size and rupture status. Stents were also sometimes used for protection of arterial branches in bifurcation aneurysms and as a bail-out option in cases of coil prolapse into the parent artery, or rarely in cases of thromboembolic complications.

When the use of stent was planned in advance, patients were given dual antiplatelet therapy (clopidogrel and acetylsalicylic acid) preoperatively, which was then continued for 6 months, and in ruptured and unexpected cases bodyweight-adjusted bolus of eptifibatide was given intravenously, followed by continuous infusion for 24 hours after which oral antithrombotics were started. Only self-expandable stents were used – Neuroform2 (Boston Scientific, Marlborough, MA, USA) in 19 patients, LEO (Balt, Montmorency, France) in 1, and Enterprise (Cordis Neurovascular/Johnson & Johnson, Miami, FL, USA) in 79 patients.

### Follow-up protocol

Our standard angiographic follow-up protocol consisted of time-of-flight magnetic resonance angiography (MRA) at 3 months after treatment, followed by digital subtraction angiography at 6 months or 1 year, decided individually according to the findings of the first exam. If control angiographic exam displayed significant recanalization or regrowth, retreatment was indicated, primarily in patients who previously suffered from bleeding. In cases of stable result without the need for retreatment, follow-up with MRA was continued on annual basis.

### Data collection and analysis

Patient’s age and sex, aneurysm location (anterior vs posterior circulation, sidewall vs bifurcation), aneurysm sac and neck size, rupture status at presentation, treatment technique (use of stent or coils alone) were noted and the differences were evaluated for statistical significance using a χ^2^ test for categorical and independent samples *t* test for numerical variables (Table 1). Additionally, aneurysms were dichotomized according to the size of the sac (small vs large) with diameter of 10 mm chosen as a threshold, and the size of the neck (narrow vs wide) with diameter of 4 mm and/or dome-to-neck ratio <2 chosen as a threshold.

**Table 1:** Baseline characteristics of aneurysms with long-term follow-up divided according to treatment technique

	Stent-assisted coiled aneurysms (N = 89)	Non-assisted coiled aneurysms (N = 194)	*P*
Patient age (years) mean ± standard deviation (range)	54.4 ± 12.11 (26-80)	51.35 ± 11.57 (12-79)	0.048^†^
Female/male, n (%)	77/12 (87/13)	150/44 (77/23)	0.101*
Aneurysm location, n (%)			
Bifurcation/sidewall	18/71 (20/80)	127/67 (65/35)	<0.001*
Anterior/posterior circulation	69/20 (77.5/22.5)	154/40 (79.4/20.6)	0.843*
Aneurysm diameter (mm) mean ± standard deviation (range)	8.8 ± 5.02 (2-22)	6 ± 3.02 (2-18)	<0.001^†^
Aneurysm size: large/small, n (%)	34/55 (38.2/61.8)	26/168 (13.4/86.6)	<0.001*
Neck diameter (mm) mean ± standard deviation (range)	4.68 ± 2.48 (1.5-16)	2.72 ± 1.25 (1-10)	<0.001^†^
Aneurysm neck: wide/narrow, n (%)	71/18 (79.8/20.2)	77/117 (39.7/60.3)	<0.001*
Clinical status: ruptured/unruptured, n (%)	20/69 (22/78)	116/78 (60/40)	<0.001*

*χ^2^ test.

†independent samples *t* test.

**Figure 1:**
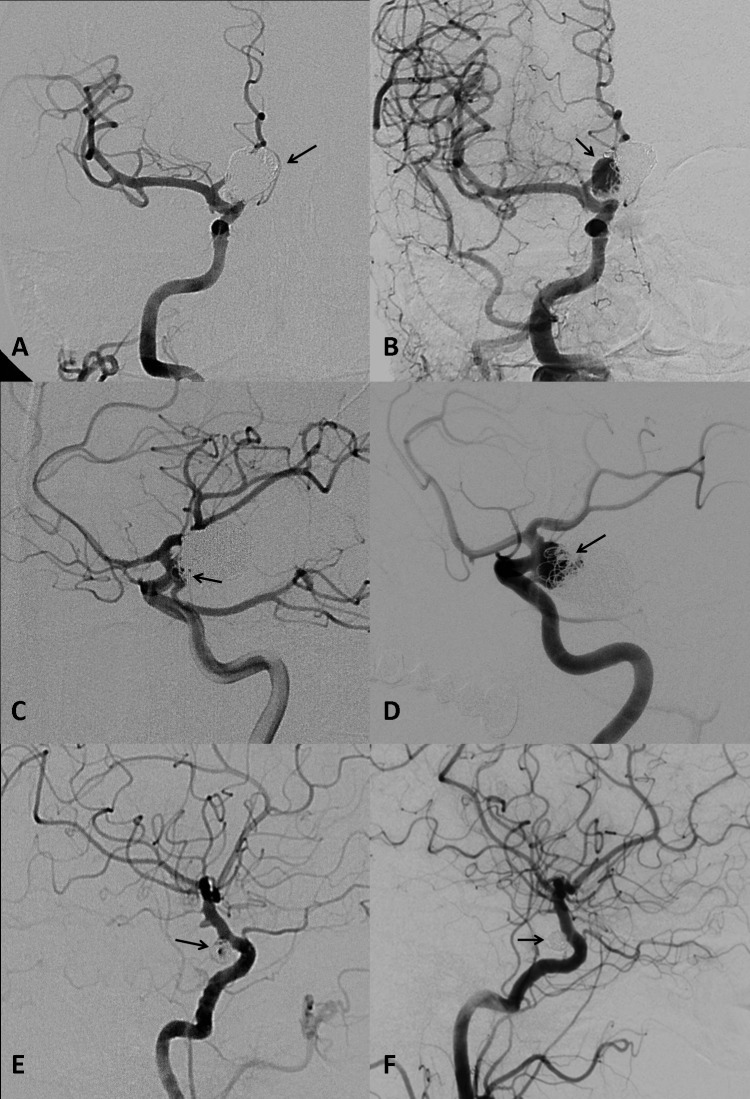
Examples of digital subtraction angiography (DSA) findings immediately after endovascular treatment of intracranial aneurysms and morphological changes occurring at 12 month follow-up. (**A,B**) Recanalization of initially completely occluded ophtalmic aneurysm (arrow) treated with non-assisted coiling. (**C,D**) Significant regrowth of residual neck of posterior communicating artery aneurysm (arrow) treated with stent-assisted coiling. (**E,F**) Delayed occlusion of residual filling of ophtalmic segment carotid aneurysm (arrow) treated with stent-assisted coiling.

Aneurysm occlusion at the end of the treatment and at the follow-up exam was classified using Roy/Raymond scale (9). Control angiographic result was described as stable (no change of value in R/R scale), recanalization (change from initial grade 1 to grade 2 or 3), regrowth (progression from grade 2 to 3 or enlargement of diameter of initial grade 3), and progressive occlusion (change from grade 2 or 3 to grade 1) (Figure 1). χ^2^ test was used to assess the differences between two treatment groups with respect to the degree of initial aneurysm occlusion and control angiographic result. Multivariate logistic regression analysis was performed to determine which of the demographic, clinical, and anatomical factors were associated with angiographic progressive occlusion, recurrence, and retreatment. Statistical tests were performed using MedCalc, version 12.7. (MedCalc Software, Ostend, Belgium). A *P* value of <0.05 was considered statistically significant. A single author (D.O.) performed all the measurements and analysis on angiographic images of the initial treatment and control exams blinded for previous findings in written reports.

## Results

There were 260 patients who had long-term control angiographic exams, with 283 treated aneurysms – 89 aneurysms in SAC group and 194 in NAC group. Significant difference between the groups was found in all parameters (Table 1), except sex (87% of female patients in SAC and 77% in NAC group, *P* = 0.101) and aneurysm location (77.5% of anterior circulation aneurysms in SAC and 79.4% in NAC group, *P* = 0.843). Aneurysms in SAC group were predominately unruptured (78%), had wide neck (79.8%), and were located on sidewall (80%), while aneurysms in NAC group were predominantly ruptured (60%), had narrow neck (60.3%), and were located on bifurcations (65%).

As initial angiographic result, R/R grade 1 was achieved in 20% of aneurysms in SAC group and in 43% in NAC group, grade 2 in 15% in SAC and 30% in NAC group, and grade 3 in 65% in SAC and 27% in NAC group, with all differences being significant (Table 2).

**Table 2:** Angiographic result at the time of coiling and after long-term follow-up divided according to treatment technique using Roy/Raymond scale (R/R) (9)

	Stent-assisted coiled aneurysms (N = 89)	Non-assisted coiled aneurysms (N = 194)	*P*
**Initial angiographic result**, n (%)			
**R/R 1**	18 (20)	84 (43)	<0.001*
**R/R 2**	13 (15)	57 (30)	0.012*
**R/R 3**	58 (65)	53 (27)	<0.001*
**Follow-up period** (months) mean ± standard deviation (range)	29.27 ± 17.17 (12-81)	34.93 ± 22.32 (12-90)	
**Control angiographic result**, n (%)			
**R/R 1**	50 (56.2)	98 (50.5)	0.449*
**R/R 2**	15 (16.8)	47 (24.2)	0.216*
**R/R 3**	24 (26.9)	49 (25.2)	0.874*

*χ^2^ test.

As control angiographic result, R/R 1 was observed in 56.2% of aneurysms in SAC group and 50.5% of aneurysms in NAC group. Grade 2 was found in 16.8% of aneurysms in SAC group and in 24.2% in NAC group. 1/4 aneurysms in both groups were residual aneurysms (26.9% in SAC and 25.2% in NAC group respectively) with no significant differences between the groups (Table 2). At follow-up, 2 of 83 implanted stents were occluded (2.4%) because both patients prematurely discontinued the use of clopidogrel due to increased bleeding and bruising.

When angiographic results were converted to descriptive terms (Table 3), significant difference between two groups existed only in the rate of progressively occluded aneurysms – there were more of these in SAC group (38 of 89 aneurysms, 42.7%) than in NAC group (46 of 194, 23.7%, *P* = 0.002). The number of aneurysms displaying stable result (35.9% vs 43.8%) and regrowth (14.6% vs 16.5%) was not significantly different, and the difference in rates of recanalization was near the level of significance (6.7% in SAC group and 15.9% in NAC group, *P* = 0.051).

**Table 3:** Morphological outcome of aneurysms with at long-term follow-up divided according to treatment technique

Morphological outcome, n (%)	Stent-assisted coiled aneurysms (N = 89)	Non-assisted coiled aneurysms (N = 194)	*P*
Recanalization	6 (6.7)	31 (15.9)	0.051*
Regrowth	13 (14.6)	32 (16.5)	0.819*
Stable result	32 (35.9)	85 (43.8)	0.264*
Progressive occlusion	38 (42.7)	46 (23.7)	0.002*

*χ^2^ test.

Multivariate logistic regression identified the use of stent (*P* = 0.015, odds ratio [OR] 2.22, 95% confidence interval [CI] 1.17-4.21) and large aneurysm size (*P* = 0.018, OR 0.42, 95% CI 0.20-0.86) as factors associated with progressive occlusion (Table 4), and large aneurysm size (*P* < 0.001, OR 3.41, 95% CI 1.71-6.79) and posterior circulation location (*P* = 0.011, OR 2.29, 95% CI 1.21-4.32) as most predictive of aneurysm recurrence (both recanalization and regrowth) (Table 5).

**Table 4:** Results of logistic regression analysis for factors influencing progressive occlusion of treated aneurysm

	Odds ratio	95% confidence interval	P*
Sex			
female	1.00		
male	0.61	0.29-1.27	0.186
Clinical status			
unruptured	1.00		
ruptured	0.66	0.36-1.21	0.182
Aneurysm location			
anterior circulation	1.00		
posterior circulation	1.36	0.70-2.66	0.361
Aneurysm location			
bifurcation	1.00		
sidewall	1.13	0.59-2.15	0.710
Aneurysm size			
small	1.00		
large	0.42	0.20-0.86	0.018
Aneurysm neck			
wide	1.00		
narrow	0.75	0.41-1.35	0.339
Coiling technique			
non-assisted	1.00		
stent-assisted	2.22	1.17-4.21	0.015

*Hosmer- Lemeshov test was 0.022.

**Table 5:** Results of logistic regression analysis for factors influencing aneurysm recurrence after treatment

	Odds ratio	95% confidence interval	P*
Sex			
female	1.00		
male	1.19	0.61-2.32	0.611
Clinical status			
unruptured	1.00		
ruptured	1.32	0.72-2.41	0.374
Aneurysm location			
anterior circulation	1.00		
posterior circulation	2.29	1.21-4.32	0.011
Aneurysm location			
bifurcation	1.00		
sidewall	0.70	0.37-1.34	0.284
Aneurysm size			
small	1.00		
large	3.41	1.71-6.79	<0.001
Aneurysm neck			
wide	1.00		
narrow	1.19	0.67-2.12	0.552
Coiling technique			
non-assisted	1.00		
stent-assisted	0.55	0.27-1.14	0.109

*Hosmer- Lemeshov test was 0.138.

Of all treated aneurysms, 10% underwent retreatment (53 out of 529), and from the group of aneurysms with long-term follow-up 46 underwent retreatment (16.3%). Eight aneurysms required multiple retreatment procedures (15.1% of all retreated) and 5 aneurysms were retreated due to rupture/rerupture (0.9% of all treated). When retreated aneurysms were divided according to initial treatment modality (Table 6), significant difference was found only in aneurysm size – there were more large aneurysms in SAC group (78.6% vs 37.5% in NAC group, *P* = 0.025). Unlike in the sample of all aneurysms, among retreated ones, large aneurysms were prevalent in SAC group and their share was much larger than in the NAC group (increase from 38.2% in SAC and from 13.4% in NAC group, Table 1). As expected, most retreated aneurysms had initial R/R grade of 3. Multivariate logistic regression identified large aneurysm size (*P* < 0.001, OR 8.34, 95% CI 3.70-18.77) and posterior circulation location (*P* = 0.029, OR 2.33, 95% CI 1.09-5.03) as most predictive of aneurysm retreatment (Table 7).

**Table 6:** Baseline characteristics and initial angiographic result of aneurysms with long-term follow-up that underwent retreatment, divided according to initial treatment technique

	Stent-assisted coiled aneurysms (N = 14)	Non-assisted coiled aneurysms (N = 32)	*P*
Aneurysm location, n (%)			
Bifurcation/sidewall	5/9 (35.7/64.3)	20/12 (62.5/37.5)	0.175^†^
Anterior/posterior circulation	8/6 (57.1/42.9)	22/10 (68.8/31.2)	0.671^†^
Aneurysm size: large/small	11/3 (78.6/21.4)	12/20 (37.5/62.5)	0.025^†^
Aneurysm neck: wide/narrow	12/2 (85.7/14.3)	16/16 (50/50)	0.051^†^
Clinical status: ruptured/unruptured	4/10 (28.6/71.4)	20/12 (62.5/37.5)	0.072^†^
Initial angiographic result			
R/R 1*	3 (21.4)	9 (28.1)	0.912^†^
R/R 2	0 (0)	6 (18.7)	0.157^‡^
R/R 3	11 (78.6)	17 (53.2)	0.194^†^

*Roy/Raymond scale (9).

†χ^2^ test.

‡Fisher exact test.

**Table 7:** Results of logistic regression analysis for factors influencing aneurysm retreatment

	Odds ratio	95% confidence interval	P*
Clinical status			
unruptured	1.00		
ruptured	1.66	0.77-3.58	0.194
Aneurysm location			
anterior circulation	1.00		
posterior circulation	2.33	1.09-5.03	0.029
Aneurysm location			
bifurcation	1.00		
sidewall	0.77	0.34-1.77	0.545
Aneurysm size			
small	1.00		
large	8.34	3.70-18.77	<0.001
Aneurysm neck			
wide	1.00		
narrow	0.98	0.47-2.06	0.967
Coiling technique			
non-assisted	1.00		
stent-assisted	0.62	0.25-1.53	0.299

*Hosmer- Lemeshov test was 0.693.

## Discussion

A major limitation of endovascular embolization of intracranial aneurysms with coils is aneurysm recurrence. It results from compaction of coils and subsequent recanalization of occluded aneurysm, and from regrowth of incompletely coiled aneurysm. Large aneurysm and neck-size and incomplete occlusion on initial angiography have been repeatedly associated with recurrence after coiling (3,4,10,11).

In an effort to improve angiographic outcome after embolization of large and wide-neck aneurysms, intracranial stents were introduced as mechanical support for coils (6,12-15) with a paradoxical result – stented aneurysms had a lower rate of immediate complete occlusion than only coiled aneurysms (16-18). Our results also displayed this trend – there was a significantly smaller percentage of completely occluded aneurysms and significantly greater percentage of aneurysms with residual filling of lumen in SAC than in NAC group. This is usually explained by more difficult manipulation of microcatheter once the stent is positioned, and by the necessary use of antiplatelet medications inhibiting the thrombus formation within coils in aneurysm sac (19).

The observed initial angiographic outcome of stented aneurysms changes during the follow-up period due to progressive thrombosis and occlusion, and independently, due to lower recurrence rate compared to only-coiled aneurysms (19-23). These two phenomena are believed to be a result of hemodynamic and biological effect of the stent on the vessel harboring the aneurysm (24). Experimental studies showed that placement of the stent across the aneurysm neck produces flow alterations (25) and induces endothelization (26), both favorable for the long-term durability of embolization.

Our results are in accordance with those described above – stented aneurysms displayed significantly higher rate of delayed occlusion and a strong trend toward the reduction of recanalization rate. Expectedly, multivariate analysis identified the use of stents and large aneurysm size as the factors most strongly associated with progressive occlusion, and large aneurysm size as the factor most predictive of recurrence. Although aneurysm location was usually not identified as having statistically significant effect on recurrence, we found that posterior circulation location to be an important predictor. Similarly, Raymond et al (3) showed that basilar bifurcation aneurysm, the most common posterior circulation location, had the highest recurrence rate, 39.4%.

Retreatment rate in our study is consistent with published data (10.3% in Ferns et al) (27) and was significantly influenced only by large aneurysm size and posterior location, the same factors determining aneurysm recurrence.

It must be noted that despite the improvement of angiographic outcome in SAC group, almost half of aneurysms in both groups were still incompletely occluded with persisting risk of rupture. Our rate of complete aneurysm occlusion (56.2% in SAC group and 50.5% in NAC group) was lower than a previously published rate (61%-71.9% for SAC and 61.5% for NAC) (27-29), but this did not lead to an increased rate of delayed rupture or re-rupture (0.9% of all coiled aneurysms in our institution).

The major limitation of this study was that it compared inhomogeneous aneurysm populations, which is reflected in significant differences between the groups in almost all evaluated parameters, especially those regarding aneurysm characteristics. Although one might expect that predominant sidewall location (80%), small aneurysm size (61.6%), and the number of narrow neck aneurysms (20.2%) in SAC group could bias the results toward its pro-occlusive effect, and that more common bifurcation location (65%) and significant percentage of wide neck aneurysms (39.7%, a consequence of dichotomization criteria) could bias the results in NAC group toward more recurrences, those effects were not evident after multivariate analysis.

Despite the mentioned shortcomings, our results provide fair representation of real life situation where operators’ choice of treatment technique is often not based solely on generally accepted indications (mainly anatomical and clinical), but is also influenced by other unrelated factors – eg, operators’ experience, difficulty of access to intracranial vasculature, local availability of materials, unexpected events/complications occurring during the procedure, etc. Nevertheless, we believe that widely accepted indications for stent-assisted coiling in large, wide-neck, sidewall and unruptured aneurysms were generally present and that use of stents was justified. However, it seems that their future use as a support to coiling in the same population of aneurysms will be limited due to promising results of flow-diverters with much higher occlusion rates (30,31).

In conclusion, our study confirmed that progressive occlusion of initially incompletely occluded aneurysms can be expected after stent-assisted coiling, but significant effect of stent on reduction of recurrence and retreatment rate was not observed. Hence, long-term angiographic follow-up is still needed for both stent-assisted and non-assisted coiled aneurysms.
